# Structural Analysis of Spermidine Synthase from *Kluyveromyces lactis*

**DOI:** 10.3390/molecules28083446

**Published:** 2023-04-13

**Authors:** Seongjin Kim, Jeong Ho Chang

**Affiliations:** 1 Department of Biology Education, Kyungpook National University, 80 Daehak-ro, Buk-gu, Daegu 41566, Republic of Korea; 2 Department of Biomedical Convergence Science and Technology, Kyungpook National University, 80 Daehak-ro, Buk-gu, Daegu 41566, Republic of Korea; 3 Science Education Research Institute, Kyungpook National University, 80 Daehak-ro, Buk-gu, Daegu 41566, Republic of Korea

**Keywords:** SpdS, spermidine, putrescine, polyamine, aminopropyltransferase

## Abstract

Spermidine is a polyamine molecule that performs various cellular functions, such as DNA and RNA stabilization, autophagy modulation, and eIF5A formation, and is generated from putrescine by aminopropyltransferase spermidine synthase (SpdS). During synthesis, the aminopropyl moiety is donated from decarboxylated S-adenosylmethionine to form putrescine, with 5′-deoxy-5′-methylthioadenosine being produced as a byproduct. Although the molecular mechanism of SpdS function has been well-established, its structure-based evolutionary relationships remain to be fully understood. Moreover, only a few structural studies have been conducted on SpdS from fungal species. Here, we determined the crystal structure of an apo-form of SpdS from *Kluyveromyces lactis* (*Kl*SpdS) at 1.9 Å resolution. Structural comparison with its homologs revealed a conformational change in the α6 helix linked to the gate-keeping loop, with approximately 40° outward rotation. This change caused the catalytic residue Asp170 to move outward, possibly due to the absence of a ligand in the active site. These findings improve our understanding of the structural diversity of SpdS and provide a missing link that expands our knowledge of the structural features of SpdS in fungal species.

## 1. Introduction

Polyamines are ubiquitous components in most cells, among which diamine putrescine, triamine spermidine, and tetraamine spermine are widely found in living organisms [1,2,3,4]. Other polyamines such as thermospermine are found in thermophiles that survive at extremely high temperatures [5,6]. Polyamines bind easily to cellular polyanions; in *Escherichia coli*, almost half of putrescine and 90% of spermidine is complexed with cellular RNA [7,8]. Spermidine is a type of polyamine produced from a shorter chain putrescine, which is involved in various biological processes, including the regulation of membrane potential, inhibition of nitric oxide synthase (NOS), and the induction of autophagy [9]. In particular, spermidine suppresses protein acetylation by inhibiting the activity of acetyltransferases such as E1A-associated protein p300 (EP300) [10], which can rapidly induce autophagy by altering autophagic flux [10]. Spermidine also influences translation through eIF5A, which forms an uncommon amino acid hypusine on eIF5A by conjugating a lysine residue and the aminobutyl moiety from spermidine [11,12].

Aminopropyltransferases are involved in synthesizing polyamines in dependence of decarboxylated S-adenosylmethionine (dcSAM), which is converted from *S*-adenosylmethionine (SAM) by *S*-adenosylmethionine decarboxylase (SAMDC) [13,14,15,16]. In turn, dcSAM facilitates the catalytic reaction by transferring its aminopropyl moiety to a shorter-chain polyamine, resulting in a longer-chain polyamine, with methylthioadenosine (MTA) formed as a byproduct [17,18].

Spermidine synthase (SpdS; EC 2.5.1.16) is a major type of aminopropyltransferase that converts putrescine into spermidine [13,14,15]. The general reaction mechanism of SpdS has been well established: putrescine initiates nucleophilic attack on dcSAM, which donates an aminopropyl moiety to active site residues, such as Asp, Tyr, and Ser, to produce spermidine [1]. There are two types of enzymatic mechanisms for SpdS: ping-pong and sequential. SpdS in *Glycine max* (soybean) and *E. coli* follow a ping-pong mechanism [19,20], whereas SpdS in *Thermotoga maritima*, *Plasmodium falciparum*, *Rattus rattus* (rat), and *Homo sapiens* utilize a sequential mechanism [1,21,22,23].

SpdS consists of an N-terminal domain, which contains four β-strands, and a C-terminal domain containing a Rossmann-like fold [22]. In addition, SpdS contains a structural feature known as the gate-keeping loop, which is located in the vicinity of the entrance to the active site [22,24]. This loop is involved in recognizing the putrescine substrate, and mutational studies have revealed that several residues in this loop contribute toward substrate-binding and stabilization of the active site [25]. Since the first crystal structure of an aminopropyltransferase was reported in 2002 from the thermophilic anaerobic bacteria *T. maritima* [22], several other SpdS structures have been made available for several species including *H. sapiens* [1], *Arabidopsis thaliana* [26], *P. falciparum* [27], *Helicobacter pylori* [28], and *E. coli* [3]; however, our understanding of the structural features and evolutionary relationships of SpdS from fungal species remains limited.

In this study, we determined the crystal structure of the fungal *Kluyveromyces lactis* SpdS (*Kl*SpdS) and compared its gate-keeping loop and active site with homologous structures. Structural analysis revealed several distinct conformational features in *Kl*SpdS. 

## 2. Results

### 2.1. Overall Structure

**Figure 1:**
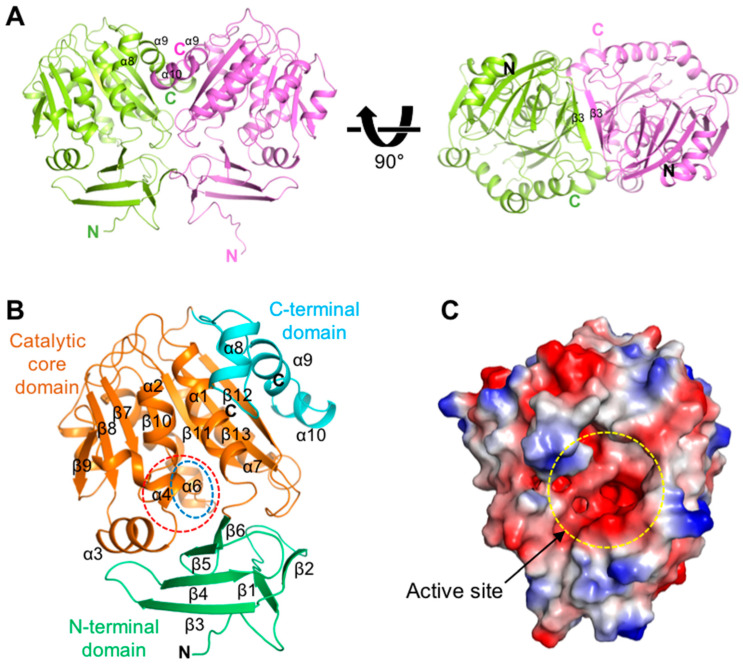
Overall structure of spermidine synthase (SpdS) from *Kluyveromyces lactis* (*Kl*SpdS). (**A**) The dimeric structure of *Kl*SpdS is shown as a cartoon. Chain A is shown in lime green, and chain B is shown in pink. Different view of 90° rotation along the *x*-axis is shown in right panel. (**B**) Monomeric structure of chain B in *Kl*SpdS. The N-terminal domain is colored green. The catalytic core domain is shown in orange. The C-terminal domain is shown in cyan. The active site of *Kl*SpdS is highlighted in the red-dashed circle. The gate-keeping loop is highlighted in the blue-dashed circle. (**C**) Electrostatic surface model of the *Kl*SpdS monomer. Red and blue represent negatively and positively charged surfaces, respectively. The active site of *Kl*SpdS is highlighted in the yellow-dashed circle.

*Kl*SpdS exists as a dimer in the asymmetric unit, and each monomer in the dimeric *Kl*SpdS is positioned with two-fold symmetry (Figure 1A). The N- and C-terminal regions in each monomer are mainly involved in dimerization via interactions with β3 and α8-α9 of their partner molecules. Each *Kl*SpdS monomer consists of three domains: an N-terminal domain (residues 4–66), a central catalytic core domain (residues 67–250), and a C-terminal domain (residues 251–292; Figure 1B, Supplemental Figure S1). The N-terminal domain includes six β-strands and is smaller than the catalytic core domain. The first two β-sheets of the N-terminal domain form a β-hairpin structure, followed by a four-stranded β-strands. Meanwhile, the catalytic core domain contains seven β-strands that form a Rossmann-like fold from β7 to β13 and seven α-helices. This canonical topology appears widely in nucleotide-binding enzymes and in class I MTases, which use dcSAM as a methyl moiety donor [24]. The C-terminal domain includes three α-helices (α8–α9) that mainly contribute toward dimer formation. The electrostatic surface representation of *Kl*SpdS revealed a large cavity in the catalytic core domain between the N- and C-terminal domains (Figure 1C); the cavity was highly negatively charged, suggesting that positively charged dcSAM bind to putrescine in this active site pocket.

### 2.2. Gate-Keeping Loop

**Figure 2:**
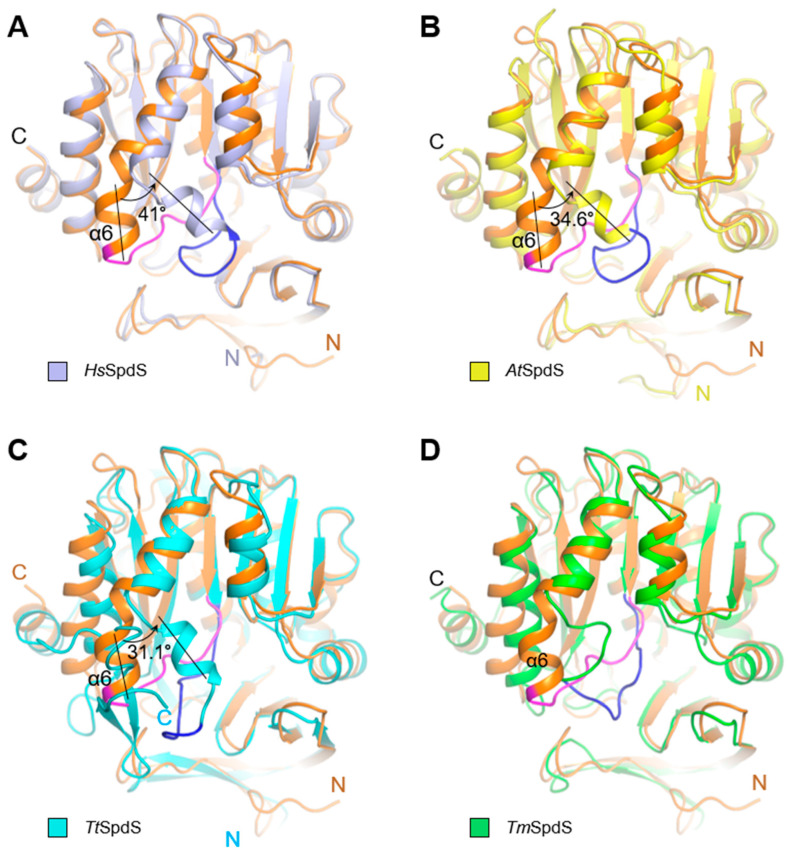
Comparisons of the gate-keeping loop between the apo structure of spermidine synthase from *Kluyveromyces lactis* (*Kl*SpdS) and four other species. Each figure represents the superposition of the monomers to compare the gate-keeping loop. In (**A**–**D**), the monomer of the apo-*Kl*SpdS is colored orange. (**A**) Superposition of apo-*Kl*SpdS with that of SpdS from *Homo sapiens* (*Hs*SpdS; PDB code 2O0L). The *Hs*SpdS monomer is shown in light blue. (**B**) Superposition of the apo-*Kl*SpdS with that of SpdS from *Arabidopsis thaliana* (*At*SpdS; PDB code 6O63). The *At*SpdS monomer is shown in yellow. (**C**) Superposition of apo-*Kl*SpdS with that of SpdS from *Thermus thermophilus* (*Tt*SpdS; PDB code 1UIR). The *Tt*SpdS monomer is shown in cyan. (**D**) Superposition of apo-*Kl*SpdS with that of SpdS from *Thermotoga maritima* (*Tm*SpdS; PDB code 1INL). The *Tm*SpdS monomer is shown in lime green. The purple color indicates the gate-keeping loop of *Kl*SpdS, wherease, the gate-keeping loops from compared structures were colored in blue.

To elucidate the conformational diversity of the gate-keeping loop, the *Kl*SpdS structure was superimposed with several homologous SpdS structures (Figure 2, Table 1). While the gate-keeping loop of *H. sapiens* SpdS (*Hs*SpdS) sterically hindered the entrance of the active site, that of *Kl*SpdS had an open conformation (Figure 2A). These conformational changes could be attributed to a short α-helix (α6) in the loop that forms on one side of the putrescine-binding region and may facilitate the accurate localization of putrescine in the active site. In *Kl*SpdS, the α6 helix kinked outward by approximately 41.0° compared to *Hs*SpdS and by approximately 34.6° compared to *A. thaliana* SpdS (*At*SpdS; Figure 2B). The gate-keeping loop in *Thermus thermophilus* SpdS (*Tt*SpdS) had a slightly different conformation compared to that of *Hs*SpdS and *At*SpdS (Figure 2C) and the α6 helix kinked inward by 31.1° compared to *Kl*SpdS. Notably, the α6 helix of *Thermotoga maritima* SpdS (*Tm*SpdS) was shorter than that of *Kl*SpdS, suggesting that its longer gate-keeping loop could be more flexible (Figure 2D).

To assess conformational changes in the gate-keeping loop upon ligand binding, the *Kl*SpdS structure was superimposed with the available structures of dcSAM complexed with *Hs*SpdS, *At*SpdS, *P. falciparum* SpdS (*Pf*SpdS), and *Tc*SpdS (Supplemental Figure S2, Table 1). The gate-keeping loop in the *Hs*SpdS-dcSAM complex was not visible due to disordered (Supplemental Figure S2A), indicating that dcSAM could open the active site in *Hs*SpdS by altering the conformation of the gate-keeping loop. Meanwhile, the α6 helix conformation of the *At*SpdS-dcSAM complex differed by approximately 35.2° compared to *Kl*SpdS (Supplemental Figure S2B). The conformations of apo-*At*SpdS and the *At*SpdS-dcSAM complex were highly similar, indicating that dcSAM binding has no significant effect on *At*SpdS conformation. The α6 helix conformations of *Pf*SpdS and *Tc*SpdS complexed with dcSAM also differed from those of *Kl*SpdS by approximately 35° (Supplemental Figure S2C,D).

Next, we investigated whether the gate-keeping loop conformation changed upon ligand binding in various species. No significant changes were observed in the gate-keeping loop conformation with binding of ligands such as MTA, adoDATO, 4MCA, putrescine, dcSAM, and spermidine in *A. thaliana*, *T. thermophilus*, *T. maritima*, or *P. falciparum*; however, changes were observed for *H. sapiens* (Supplemental Figure S3). *Tt*SpdS-MTA, *Tm*SpdS-adoDATA, and *At*SpdS-4MCHA complexes shared similar gate-keeping loop conformations. Although the α6 helix induced fit upon ligand binding in *Tc*SpdS, no conformational changes in the gate-keeping loop were observed in the other four species (Supplemental Figures S2 and S3). When we compared the structures of *Hs*SpdS and *Pf*SpdS in complex with putrescine, dcSAM, spermidine, and MTA, the gate-keeping loops exhibited almost the same conformation, except for those in the MTA complexes, which had a transition angle of 9.6° (Supplemental Figure S4). This might be attributed to the residues joining this region, which were nearly the same except for Ile201 in *Pf*SpdS instead of Met178 in *Hs*SpdS. However, since both amino acids have a non-polar character, the conformational differences might not be substantial.

### 2.3. Active Site

**Figure 3:**
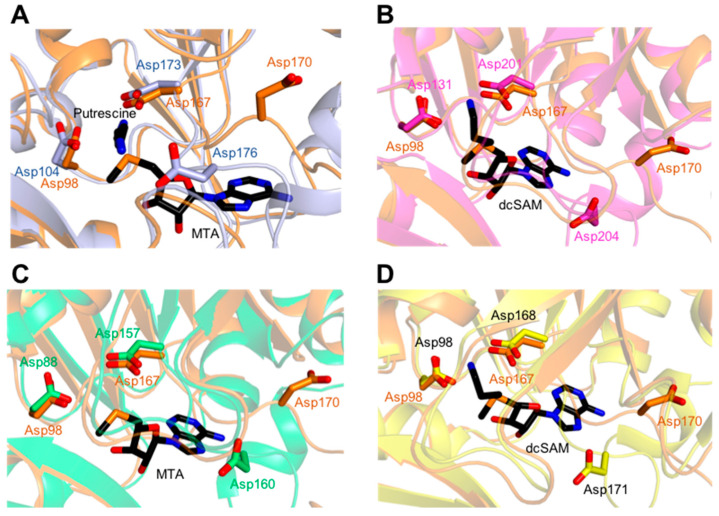
Structural comparisons of the three key aspartic residues in the active site of spermidine synthase (SpdS) from five different species. In (**A**–**D**), the *Kluyveromyces lactis* SpdS (*Kl*SpdS) monomer is shown in orange. (**A**) The *Homo sapiens* SpdS (*Hs*SpdS) monomer is shown in light blue. (**B**) The *Arabidopsis thaliana* SpdS (*At*SpdS) monomer is shown in warm pink. (**C**) The *Plasmodium falciparum* SpdS (*Pf*SpdS) monomer is shown in lime green. (**D**) The *Trypanosoma cruzi* SpdS (*Tc*SpdS) monomer is shown in yellow.

The catalytic residues Asp98, Asp167, and Asp 170 of *Kl*SpdS were highly conserved in other SpdS structures (Figure 3). Asp98 captures the aminopropyl moiety of dcSAM and remains ready for the initiation of nucleophilic attack by putrescine. Asp167 plays a crucial role in the deprotonation of putrescine, while Asp170 is required for accurate putrescine binding. To examine the possible active site of *Kl*SpdS, its structure was superposed with those of *Hs*SpdS, *At*SpdS, *Pf*SpdS, and *Tc*SpdS complexed with specific ligands (Figure 3A–D). Although Asp98 and Asp167 had conformations similar to the other structures, conformation of Asp170 was distinct, possibly due to changes caused by ligand binding.

**Figure 4:**
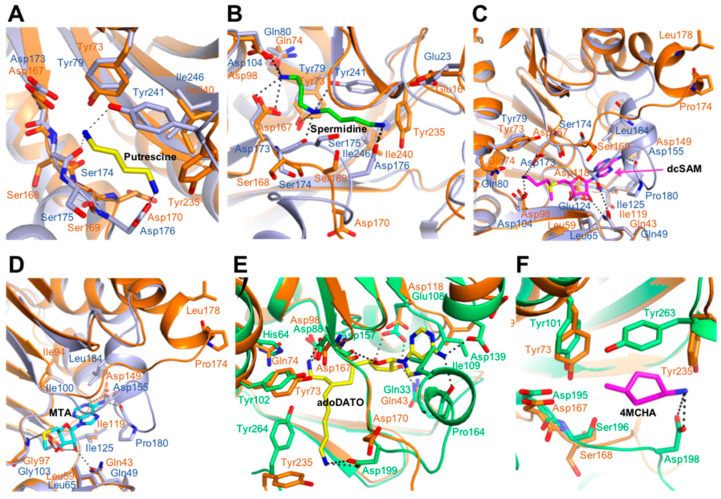
Superposition of the *Kl*SpdS structure with SpdS-ligand complexes. (**A**) Superposition of the structures of spermidine synthase (SpdS) from *Kluyveromyces lactis* (*Kl*SpdS) and the *Homo sapiens* (*Hs*SpdS)–putrescine complex. The substrate putrescine is shown in yellow. The panel provides a detailed view of the interaction between putrescine and *Kl*SpdS, with overlaid *Hs*SpdS. *Kl*SpdS and *Hs*SpdS residues are shown in orange and light blue, respectively. (**B**) Superposition of the structures of *Kl*SpdS and the *Hs*SpdS–spermidine complex. The product spermidine is shown in green. The panel provides a detailed view of the interaction between spermidine and *Kl*SpdS, with overlaid *Hs*SpdS. *Kl*SpdS and *Hs*SpdS residues are shown in orange and light blue, respectively. (**C**) Superposition of *Kl*SpdS and the *Hs*SpdS–dcSAM complex structures. The cofactor dcSAM is shown in magenta. The panel provides a detailed view of the interaction between dcSAM and *Kl*SpdS, with overlaid *Hs*SpdS. *Kl*SpdS and *Hs*SpdS residues are shown in orange and light blue, respectively. Abbreviations: dcSAM, decarboxylated *S*-adenosylmethionine. (**D**) Superposition of *Kl*SpdS and the *Hs*SpdS–MTA complex structures. The byproduct MTA is shown in cyan. The panel provides a detailed view of the interaction between MTA and *Kl*SpdS, with overlaid *Hs*SpdS. *Kl*SpdS and *Hs*SpdS residues are shown in orange and light blue, respectively. Abbreviations: MTA, methylthio-adenosine. (**E**) Superposition of *Kl*SpdS and the *Pf*SpdS–adoDATO complex structures. The inhibitor adoDATO is shown in yellow. The panel provides a detailed view of the interaction between adoDATO and *Kl*SpdS, with overlaid *Pf*SpdS. *Kl*SpdS and *Pf*SpdS residues are shown in orange and lime green, respectively. Abbreviations: adoDATO, *S*-adenosyl-1,8-diamino-3-thiooctane. (**F**) Superposition of *Kl*SpdS and the *Pf*SpdS–4MCHA complex structures. The inhibitor 4MCHA is shown in magenta. The panel provides a detailed view of the interaction between 4MCHA and *Kl*SpdS, with overlaid *Pf*SpdS. *Kl*SpdS and *Pf*SpdS residues are shown in orange and lime green, respectively. Abbreviations: 4MCHA, *trans*-4-methylcyclohexylamine.

Overall, most ligand-interacting residues were similar in the structures of *Hs*SpdS and *Kl*SpdS complexed with putrescine substrate, with the Tyr73, Asp167, and Ile240 residues in *Kl*SpdS aligning especially well with the corresponding Tyr79, Asp173, and Ile246 residues in *Hs*SpdS (Figure 4A). Putrescine generated four hydrogen bonds with the amino acids present in the active site, including three residues in the gate-keeping loop. With spermidine, most residues were well matched except for Ser174, Ser175, Asp176, and Try241 (Figure 4B), and it was stabilized by six of the seven possible hydrogen bonds between the gate-keeping loop and the active site. With the cofactor dcSAM, most residues aligned well except for Glu124, Pro180, and Leu184 (Figure 4C). Similarly, most residues were matched when bound to the MTA byproduct, except for Glu124, Pro180, and Leu184 (Figure 4D). In particular, Glu124 in *Kl*SpdS corresponded to Asp118 in *Hs*SpdS, both are negatively charged, suggesting that there would be no critical change in enzyme activity. Superpositions between the *Pf*SpdS enzyme–inhibitor complex and apo-*Kl*SpdS structures (Figure 4E,F) revealed that the residues of *Pf*SpdS did not align well with those of *Kl*SpdS in SpdS–adoDATO complexes compared to *Hs*SpdS. However, all residues in SpdS–4MCHA complex corresponded for *Hs*SpdS as well as *Pf*SpdS. Taken together, these findings suggest that SpdS exhibits different inhibitory effects when complexed with adoDATO and 4MCHA.

## 3. Discussion

Spermidine is produced from putrescine by SpdS. Although the molecular mechanism underlying SpdS function is well-established, its structure-based evolutionary relationships remain to be fully understood; moreover, very few structural studies have been conducted on SpdS from fungal species. Here, we found that the first structure of fungal *Kl*SpdS, which was determined at 1.9 Å resolution, exhibited highly similar to that of *Hs*SpdS, suggesting that *Kl*SpdS is phylogenetically closer to *Hs*SpdS than SpdS from *E. coli* and could therefore utilize a sequential mechanism rather than a ping-pong mechanism [19,20,21,22,23]. In *Kl*SpdS, the carboxylate group of Asp167 plays a major role in substrate deprotonation along with the aid of the backbone carbonyl of Ser168 and hydroxyl groups of conserved residues Tyr73 and Tyr235 [1,29]. Meanwhile, the carboxylate group of Asp170 plays an essential role in putrescine binding by anchoring the end of the diamine [1,27], whereas the carboxylate group of Asp98 is involved in binding the *N*^1^ atom of spermidine to the aminopropyl group of dcSAM [1,26]. Asp98 also promotes the initiation of nucleophilic attack on dcSAM by anchoring the aminopropyl group and fixing it in an appropriate position to initiate the enzymatic reaction [1,22].

In general, the gate-keeping loop plays a crucial role in the enzymatic reaction of SpdS through three distinct modes of action [3,22,30]. Firstly, the loop covers the active site of SpdS. Superposition of complexed *Hs*SpdS and apo-*Kl*SpdS revealed that the gate-keeping loops have distinct conformations depending on ligand binding status. Secondly, gate-keeping loops are important for substrate recognition in SpdS. For instance, the substrate specificity of SpdS can be altered through site-directed mutations of the proline residue in the gate-keeping loop (corresponding to Pro174 in *Kl*SpdS) of *E. coli* [25]. Finally, the gate-keeping loop stabilizes the active site by adopting a closed conformation. After the substrate binds to the active site, the conformation of the gate-keeping loop is changed through a series of enzymatic processes [1,24]. The gate-keeping loop was disordered in the apo structures of *Ce*SpdS and *Tc*SpdS but was well-ordered in the structures of *Pf*SpdS complexed with adoDATO, dcSAM, and dcSAM–4MCHA [7,27,29].

Taken together, the analyses of the crystal structure of *Kl*SpdS performed in this study provide insights into the structural diversity of SpdS. Despite these important findings, further studies are required to investigate two key aspects related to *Kl*SpdS. First, structural and functional studies with various ligands are essential to reveal the reaction mechanism of *Kl*SpdS. In addition, studies of SpdS from other fungal species are required to understand their molecular structure-based phylogenetic relationships with SpdS homologs.

## 4. Materials and Methods

### 4.1. Preparation of KlSpdS Expression Constructs

The gene encoding *Kl*SpdS (NCBI ID: XP_451945) was amplified from *K. lactis* genomic DNA (Korean Collection for Type Cultures, Daejeon, Republic of Korea) using polymerase chain reaction (PCR), as described previously [31]. All amplified fragments were digested using NdeI and XhoI restriction enzymes (R006S and R007S, respectively; Enzynomics, Republic of Korea) in a heating block at 37 °C for 4 h. The digested fragments were ligated with the pET28a and pET26b vectors using T4 ligase (M0202S; Roche, Germany) overnight at 18 °C to insert a hexahistidine (His6)-tag at either the N- or C-terminus of the target protein. The resulting vectors were subsequently transformed into the *E. coli* strain DH5α using kanamycin (AppliChem, Darmstadt, Germany) as a selection marker. The transformants were confirmed by colony PCR. All oligonucleotide primers used in this study were purchased from Cosmo Genetech (Seoul, Republic of Korea).

### 4.2. Purification of Recombinant Proteins

Plasmids encoding the *Kl*SpdS protein were transformed into *E. coli* strain BL21 (DE3) Star. Cells were grown at 37 °C in Luria–Bertani medium (Ambrothia, Republic of Korea) containing 50 mg/L kanamycin (AppliChem) to an optical density at 600 nm (OD_600_) of approximately 0.6. Following induction with 0.3 mM isopropyl β-D-1-thiogalactopyranoside (IPTG; Calbiochem, Germany), the cells were further grown for 16 h at 20 °C, harvested by centrifugation at 3000 rpm at 4 °C for 20 min, and resuspended in a buffer containing 20 mM Tris (pH 8.0; Sigma–Aldrich, St. Louis, MO, USA), 250 mM NaCl (AppliChem), 5% glycerol (Affymetrix, Santa Clara, CA, USA), 0.2% Triton X-100 (Sigma–Aldrich), 10 mM β-mercaptoethanol (BioBasic, Markham, ON, Canada), and 0.2 mM phenylmethylsulfonyl fluoride (Sigma–Aldrich). Next, cells were disrupted by ultrasonication (VCX-500/750, Sonics, Newtown, CT, USA) with 3-s pulse-on and 3-s pulse-off cycles continuously for 15 min. Cell debris was removed by centrifugation at 13,000 rpm for 40 min, and the supernatant was bound to Ni–NTA agarose (Qiagen, Hilden, Germany) at 7 °C for 90 min. After washing with His-binding buffer (300 mM NaCl, 50 mM Tris, pH 8.0) containing 5 mM imidazole (Sigma–Aldrich), bound proteins were eluted with His-elution buffer (200 mM NaCl, 50 mM Tris, pH 8.0) containing 250 mM imidazole (Sigma–Aldrich). Purified proteins were subjected to size-exclusion chromatography (SEC) using a HiPrep 16/60 Sephacryl S-300 HR column (GE Healthcare, Chicago, IL, USA) and an eluent buffer containing 20 mM Tris (pH 7.5), 150 mM NaCl, and 2 mM dithiothreitol (DTT; Calbiochem). Following SEC, proteins were stored at −80 °C until crystallization. Protein purity was assessed by performing sodium dodecyl sulfate-polyacrylamide gel electrophoresis using a 15% acrylamide gel, which produced a single band corresponding to the calculated molecular weight of the target protein.

### 4.3. Crystallization and Improvements

All crystallization experiments were performed at 20 °C using the sitting-drop vapor diffusion method in 96-well sitting-drop plates (Art Robbins Instruments, Sunnyvale, CA, USA). Approximately 600 different conditions from sparse-matrix screening solution kits were tested to identify the optimal crystallization conditions. The following kits were used: PEG/Ion (HR2-126 and −098), Index (HR2-144), Salt Rx 1/2 (HR2-107 and -109), and Crystal Screen 1/2 (HR2-110 and -112) from Hampton Research (Viejo, CA, USA), Wizard 1/2 (CS-311, Jena Bioscience, Germany), and SG1 Screen (MD1-88, Molecular Dimensions, Rotherham, UK). *Kl*SpdS crystals grew within 24 h in drops containing equal volumes (1 μL) of protein sample (10 mg/mL in 150 mM NaCl, 2 mM DTT, and 20 mM Tris, pH 7.5) and reservoir solution (9.2% *v/v* TacsimateTM pH 5.0, 16.5% *w/v* PEG 3350). Additional screening was performed using additive (HR2-428, Hampton Research) and detergent (HR2-406, Hampton Research) screening kits. The optimal crystallization conditions used 9.2% *v/v* TacsimateTM (pH 5.0), 16.5% (*w*/*v*) PEG 3350, and 2.5% (*v*/*v*) 1-butanol.

### 4.4. Data Collection and Structure Determination

Prior to data collection, 30% glycerol was added to the reservoir solutions as a cryoprotectant, and crystals were flash-cooled in liquid nitrogen. All diffraction datasets were collected at 100 K on a beamline 5C at the Pohang Accelerator Laboratory (PAL, Republic of Korea) using a Quantum 270 CCD detector (USA). Data were processed using the HKL–2000 software suite (HKL Research, Charlottesville, VA, USA).

Experimental electron density maps were obtained by molecular replacement methods in Phenix software version 1.9 (Phenix Software International, Berkeley, CA, USA) and interpreted using the WinCoot program with Homo sapiens SpdS (*Hs*SpdS; PBD code, 2O06) as a search model [32,33]. The details of data collection and the statistics used in this study are listed in Table 2.

## Figures and Tables

**Table 1:** Structural similarity comparison for homologous structures of spermidine synthase among five species using Dali *^a^*.

Species	*Z*-Score	RMS Deviation (Å)	Identity (%)	C_α_	PDB Code
*Homo sapiens*	43.3	0.9	57	289	2O06
*Plasmodium falciparum*	40.6	1.4	48	270	2HTE
*Arabidopsis thaliana*	40.4	1.4	49	285	1XJ5
*Caenorhabditis elegans*	40.1	1.1	56	276	2B2C
*Trypanosoma cruzi*	40.0	1.5	44	294	4YUV

*^a^* This server computes optimal and suboptimal structural alignments between two protein structures using the DaliLite-pairwise option. Available online: http://ekhidna.biocenter.helsinki.fi/dali/ (accessed on 5 September 2019).

**Table 2:** Data collection and refinement statics for *Kl*SpdS.

Statistics	*Kl*SpdS
**Data collection**	
Space group	*P*2_1_2_1_2_1_
Cell dimensions (Å)	
a, b, c (Å)	65.252, 98.180, 102.134
α, β, γ (°)	90, 90, 90
Resolution range (Å) ^a^	50.0–1.9
No. of reflections	676,985
No. of unique reflections	52,396
*R*_merge_ ^b^ (%)	13.5 (45.2)
*I*/σ (*I*)	33.0 (4.9)
Completeness (%)	100 (100)
Redundancy	12.9 (12.1)
CC_1/2_	0.996 (0.965)
**Structure refinement**	
Resolution (Å)	48.0–1.9
No. of reflections	676,985 (52,396)
*R*_work_ ^c^/*R*_free_	16.8/19.9
No. atoms	
Protein	4639
Water	555
R.m.s. deviation	
Bond lengths (Å)	0.007
Angles (°)	0.818
Average *B*-factor (Å^2^)	23.7
Ramachandran plot (%)	
Favored region	97.4
Outliers	0.0
PDB code	8IYI

^a^ Numbers in parentheses are statistics from the highest resolution shell. ^b^ *R*_merge_ = Σ |*I*_obs_ − *I*_avg_|/*I*_obs_, where *I*_obs_ is the observed intensity of individual reflections and *I*_avg_ is the average over symmetry equivalents. ^c^ *R*_work_ = Σ ||*F*_o_| − |*F*_c_||/Σ |*F*_o_|, where |*F*_o_| and |*F*_c_| are the observed and calculated structure factor amplitudes, respectively. *R*_free_ was calculated using 5% of the data.

## Data Availability

Not applicable.

## Supplementary Materials

The following supporting information can be downloaded at: https://www.mdpi.com/article/10.3390/molecules28083446/s1.

## Sample Availability

Samples of the compounds are not available from the authors.
