# Supplementary material for: Structural Analysis of Spermidine Synthase from Kluyveromyces lactis
Source: Molecules. 2023 Apr 13;28(8):3446. doi: 10.3390/molecules28083446 (PMC10146546; doi:10.3390/molecules28083446)
Supplement: Supplementary file 1 [file molecules-28-03446-s001.zip › molecules-2317825-supplementary.pdf]

<u>Supplementary Material</u>

Structural Analysis of Spermidine Synthase from Kluyveromyces lactis

Seongjin Kim 1 and Jeong Ho Chang 1,2,3,*

1 Department of Biology Education, Kyungpook National University, 80 Daehak-ro, Buk-gu, Daegu 41566, Republic of Korea

2 Department of Biomedical Convergence Science and Technology, Kyungpook National University, 80 Daehak-ro, Buk-gu, Daegu 41566, Republic of Korea

3 Science Education Research Institute, Kyungpook National University, 80 Daehak-ro, Buk-gu, Daegu 41566, Republic of Korea

* Correspondence: jhcbio@knu.ac.kr; Tel.: +82-53-950-5913; Fax: +82-53-950-6809

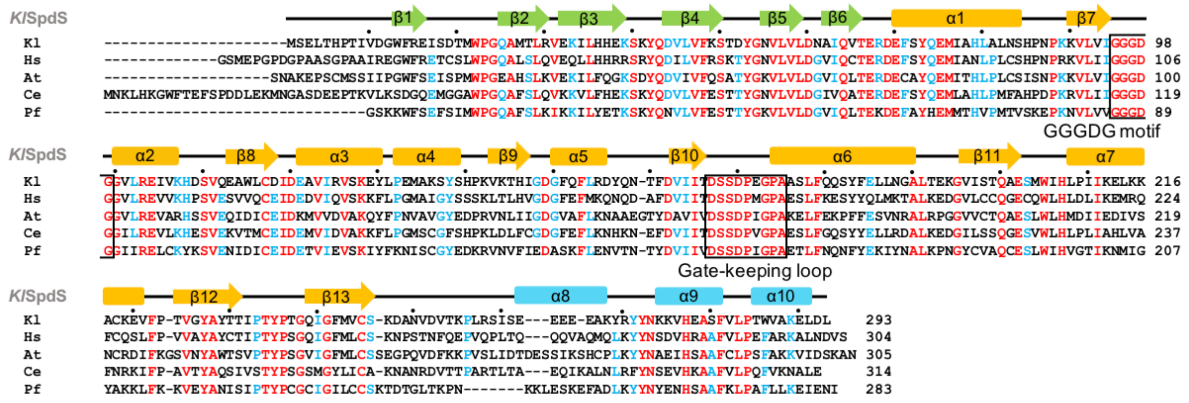

Supplemental Figure S1. Structure-based amino acid sequence alignment of spermidine synthase (SpdS) from *Kluyveromyces lactis* (*Kl*SpdS) with other homologs. Residues with 100% identity are shown in red. Residues with 80% identity are shown in cyan. Black stars indicate three critical aspartic residues (Asp 98, Asp 167, and Asp 170 in KlSpdS) involved in the aminopropyltransferase reaction. Abbreviations: *Hs: Homo sapiens. At: Arabidopsis thaliana. Ce: Caenorhabditis elegans. Pf: Plasmodium falciparum.*

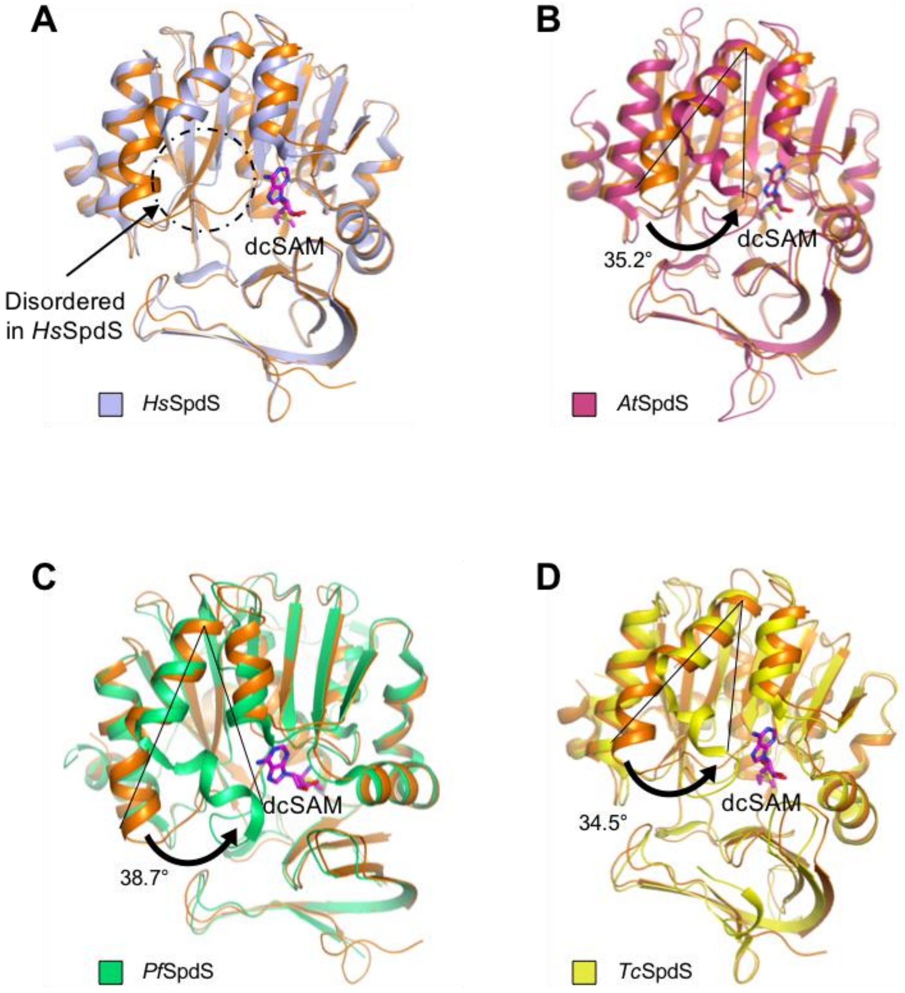

Supplemental Figure S2. Comparison of the gate-keeping loop between the apo structures of spermidine synthase (SpdS) from *Kluyveromyces lactis* (*Kl*SpdS) and SpdS complex structures from four other species. Each figure represents monomer superposition to compare the gate-keeping loop. In (**A**)–(**D**), the monomer of apo-*Kl*SpdS is shown in orange. (**A**) Superposition of apo-*Kl*SpdS and the SpdS complex structures from *Homo sapiens* (*Hs*SpdS; PDB code 2O06). The monomer of apo-*Hs*SpdS is shown in light blue. (**B**) Superposition of apo-*Kl*SpdS and the SpdS complex structures from *Arabidopsis thaliana* (*At*SpdS; PDB code 6O65). The monomer of apo-*At*SpdS is shown in warm pink. (**C**) Superposition of apo-*Kl*SpdS and the SpdS complex structures from *Plasmodium falciparum* (*Pf*SpdS; PDB code 2HTE). The monomer of apo-*Pf*SpdS is shown in lime green. (**D**) Superposition of apo-*Kl*SpdS and the SpdS complex structures from *Trypanosoma cruzi* (*Tc*SpdS; PDB code 4YUV). The monomer of apo-*Tc*SpdS is shown in yellow.

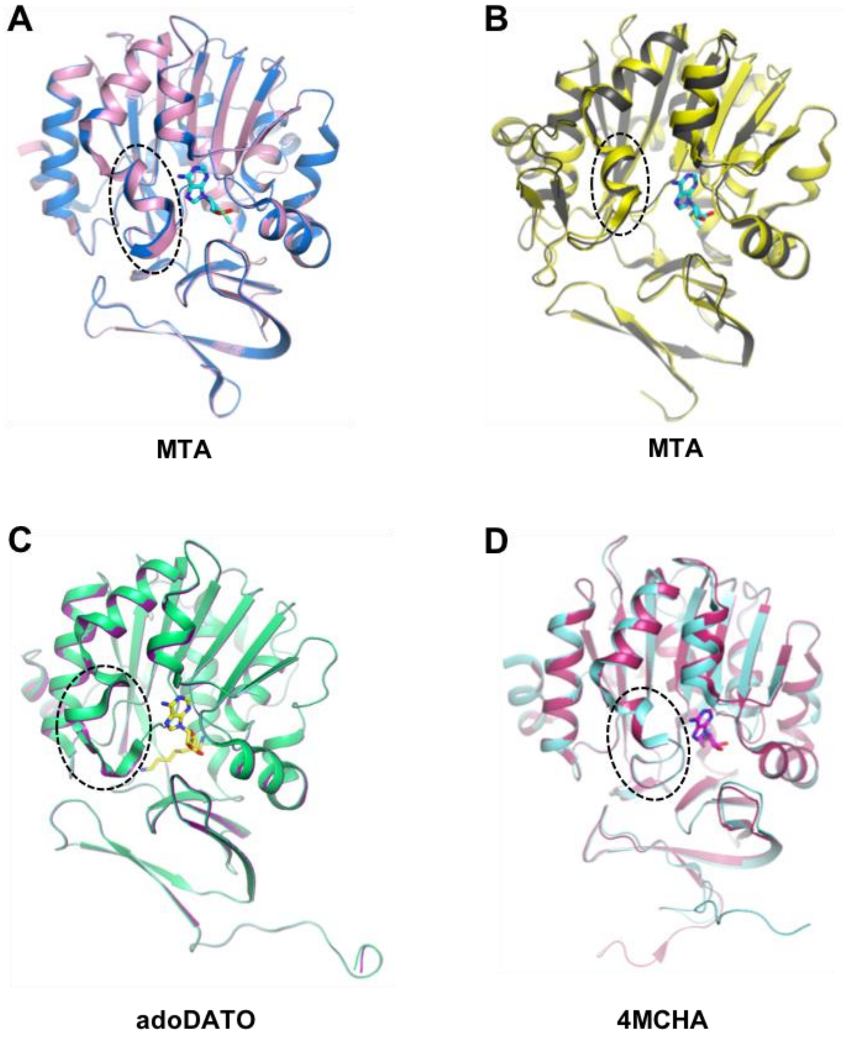

Supplemental Figure S3. Comparisons of the gate-keeping loops between apo structures of spermidine synthase (SpdS) and SpdS-substrate complex structures from four species. (**A**) Superposition of apo-SpdS (PDB code 2O0L) and the SpdS (PDB code 2O05) complex in *Homo sapiens.* The apo monomer is shown in marine and that of the complex structure is shown in pink. (**B**) Superposition of apo-SpdS (PDB code 1UIR) and the complex structure of SpdS (PDB code 3ANX) in *Thermus thermophilus.* The apo monomer is shown in yellow and that of the complex structure is shown in dark gray. (**C**) Superposition of the apo-SpdS (PDB code 1INL) and the complex structure of SpdS (PDB code 1JQ3) in *Thermotoga maritima.* The apo monomer is shown in lime green and that of the complex structure is shown in purple. (**D**) Superposition of apo-SpdS (PDB code 6O63) and the complex structure of SpdS (PDB code 6O65) in *Arabidopsis thaliana.* The apo monomer is shown in warm pink and that of the complex structure is shown in aquamarine.

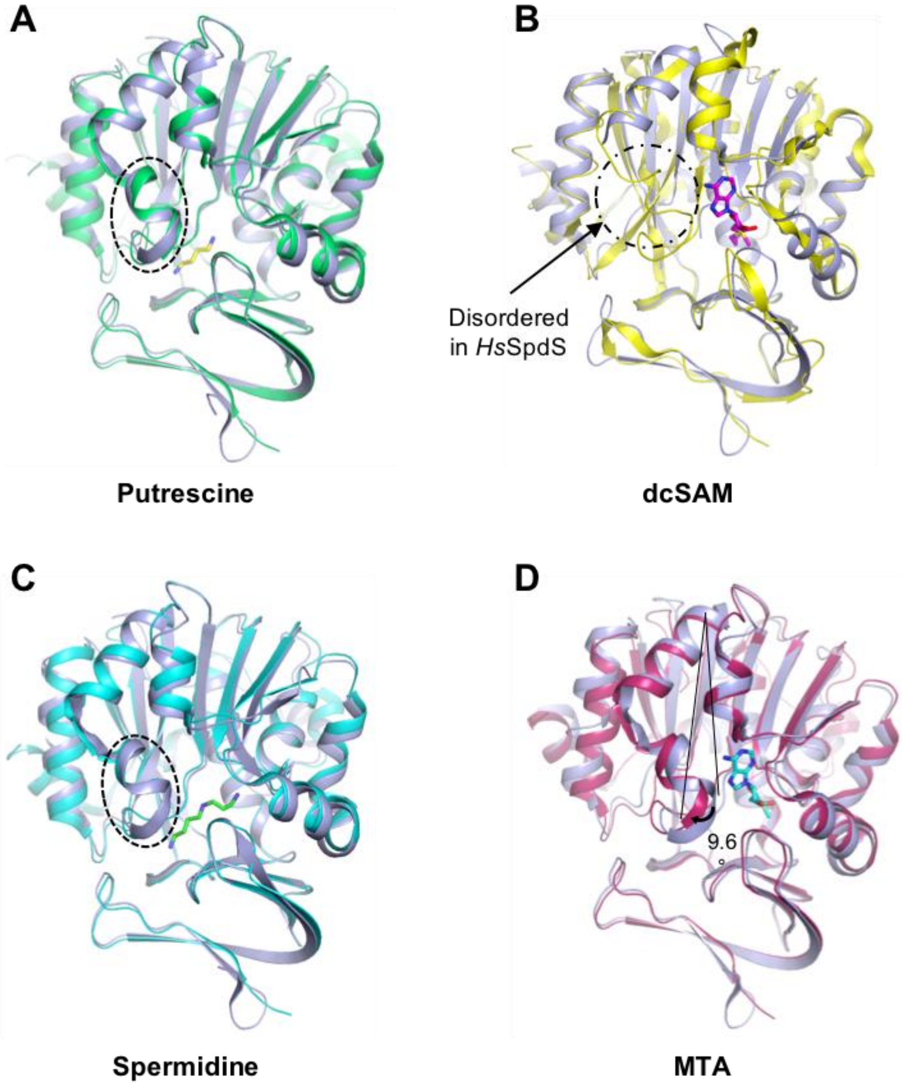

Supplemental Figure S4. Comparison of the gate-keeping loop in spermidine synthase (SpdS) complex structures from different species. In (**A**) to (**D**), the monomer of the *Hs*SpdS complex structure is shown in light blue. (**A**) Superposition of putrescine complex of *Hs*SpdS (PDB code 2O06) and *Pf*SpdS (PDB code 2HTE). The monomer of the *Pf*SpdS complex is shown in lime green. (**B**) Superposition of the dcSAM complex of *At*SpdS (PDB code 6O65) and *Pf*SpdS (PDB code 2PT6). The monomer of the *Pf*SpdS complex is shown in yellow. (**C**) Superposition of the spermidine complex of *Hs*SpdS (PDB code 2O07) and *Pf*SpdS (PDB code 2PWP). The monomer of the *Pf*SpdS complex is shown in cyan. (**D**) Superposition of the MTA complex of *Hs*SpdS (PDB code 2O05) and *Pf*SpdS (PDB code 4BP1). The monomer of the *Pf*SpdS complex is shown in warm pink.
